# Employees' Emotional, Cognitive, and Behavioral Responses to Increasing Statutory Retirement Ages

**DOI:** 10.1155/2021/6645271

**Published:** 2021-10-06

**Authors:** Jaap Oude Mulders, Kène Henkens, Hendrik P. van Dalen

**Affiliations:** ^1^Netherlands Interdisciplinary Demographic Institute (NIDI-KNAW)/University of Groningen, Netherlands; ^2^University Medical Center Groningen, Netherlands; ^3^Department of Sociology, University of Amsterdam, Netherlands; ^4^Tilburg School of Economics and Management (TISEM), Tilburg University, Netherlands

## Abstract

Increasing statutory retirement ages around the world are forcing employees to prolong their working lives. We study the different ways in which mid- and late-career workers respond to such changes. We distinguish between negative emotions about working longer, cognitive engagement with prolonged employment, and proactive behavior to facilitate longer working lives. We analyze data from 1,351 employees aged 40-66 from the Netherlands. We estimate a structural equation model to identify in which ways experiences of age discrimination, accessibility of accommodative HR facilities, and social norms in the workers' social networks are related to the three different types of responses. Results show that when employees do not experience age discrimination, when their employer offers easily accessible accommodative HR facilities, and the social norms support prolonged employment, employees have fewer negative emotional reactions and are more likely to behaviorally respond to facilitate longer working lives. When these contexts are misaligned, the reverse is generally found. We also find socioeconomic differences in the ways employees respond to the prospect of prolonged employment. This study shows the importance of supportive contexts at different levels—societally, in organizations, and in individuals' own lives—for policy changes such as increasing statutory retirement ages to be effective. Different responses between different socioeconomic groups may lead to growing long-term inequality.

## 1. Introduction

As a result of population aging, governments in developed countries are trying to promote longer working lives of their citizens. Prolonged employment is considered imperative for maintaining the financial sustainability of social security systems and public finances in general [1]. The main drivers of higher retirement ages and prolonged employment have been labor market and pension system reforms [2]. For example, many countries have closed off financially attractive routes into early retirement, transformed defined benefit pension schemes to notional defined contribution or point schemes, and increased statutory retirement ages or eligibility ages for retirement benefits [1, 3].

While these changes have contributed to increased labor force participation at older ages at the societal level, much less is known about how older workers at the microlevel respond to pension reforms that promote prolonged employment and how individual differences in responses can be accounted for. For instance, research of recent date that has focused on the effects of increasing retirement ages shows the potential of looming social and economic inequalities at higher ages. The inequalities may be a result of the different health consequences [4, 5] and the ability to cope with mental and physical challenges, especially in physically demanding jobs [6]. But the inequalities can also be generated by the lack of information or awareness of pending policy reforms. Holman et al. [7] show the lagging awareness of women in the UK of the fact that the state pension age for women would be increased from 60 to 65 and how this differs strongly by socioeconomic backgrounds.

To understand the effects of policy changes on individual workers, it is crucial to consider how they affect the hearts and minds of older workers as well as their proactive intentions to work longer [8, 9]. Most earlier research has focused on the effects of longer careers on one-dimensional outcome variables, such as emotional reactions [10] or mental health problems [11], indicating worries and negative emotions such as anger in response to the increasing statutory retirement age. Much less is known about to what extent pension reforms that stipulate higher retirement ages induce workers' cognitive engagement and proactive behaviors that enable sustainable and longer careers.

To further increase our understanding of how people respond to policy reforms that directly affect the expected duration of their working life, we argue that it is important to *simultaneously* consider different dimensions of individuals' responses. Therefore, in this study, we distinguish between emotional, cognitive, and behavioral responses of employees in reaction to an increasing statutory retirement age and study to which extent they apply these strategies. More specifically, we consider negative emotional responses, thoughts about how to remain employable until the increased retirement age, and proactive behaviors that could lead to successful longer working lives. To account for individual differences in responses, we look at how three types of restrictive circumstances, that is, in the labor market, within organizations, and in employees' social environments, are related to employees' responses, while controlling for personal factors such as age, sex, education, and income.

We study these relationships with survey data collected among a representative sample of Dutch mid- and late-career employees (40-66 years of age). The Netherlands has been characterized as being at the forefront of extending working lives and promoting active labor market participation of older individuals [1, 12], with one of the most pronounced increases in average age of retirement in recent decades [13]. The reform that provides the context for the current study is the increasing statutory or normal retirement age. This age, at which mandatory retirement is commonly applied for employees and after which individuals receive a flat rate state pension [14], was set to 65 from its inception in 1956 until 2012. Since 2013, it has been increasing gradually, and the rate of increase has been subject to intense public and political debate. According to the current rules, the statutory retirement age will reach 67 in 2024 and after that be tied to increases in the life expectancy at age 65, so that every 1-year increase in life expectancy at age 65 implies an 8-month increase in the statutory retirement age [15]. According to the Dutch public pension law, the statutory retirement age of each cohort will be definitively determined five years in advance of them reaching the statutory retirement age. According to current life expectancy projections, the statutory retirement age will be 68 years for the cohort born in 1970 and 69 years and 6 months for the cohort born in 1990. The pension law does not allow for the possibility of lowering the statutory retirement age in case the average life expectancy (at age 65) decreases.

There are two main contributions of this study to the literature. First, this is to the best of our knowledge the first study to take a comprehensive approach by developing and testing a three-factor model of negative emotions about working longer, active thinking about working longer in a durable manner, and proactive behaviors to benefit longer working lives among mid- and late-career employees. Studying these different dimensions of individual responses simultaneously increases our understanding of the myriad of ways in which employees respond to policy reforms that aim to extend their working lives. Second, we study how responses of workers can be accounted for by considering the role of relevant drivers of sustainable careers in different dimensions, such as the labor market, the workplace, and individuals' social networks [16, 17]. More specifically, we consider the roles of having experienced age discrimination in the labor market (e.g., [18]), the accessibility of accommodative HR facilities within their organization (e.g., [19]), and the social norms in the employees' social environment regarding timing of retirement and making a career switch to maintain employability throughout one's career (e.g., [20]), while controlling for personal and socioeconomic characteristics of the employees. Understanding how such varied factors are related to employees' responses can inform employers, employees, and policy makers in future policy reforms.

## 2. Theoretical Background

Impactful contextual changes or events, such as an increasing statutory retirement age, are likely to evoke emotional, cognitive, and behavioral responses [21]. As new information becomes available, people develop ways of responding or adapting to a new situation, although people sometimes choose to remain ignorant about the consequences of contextual changes. Information seeking theory [22] postulates that information about such contextual changes or events can be categorized as altering people's affect (inducing positive or negative emotions), cognition (ability to comprehend and anticipate reality), and behavior (ability to make decisions). Here, we apply this perspective to employees' responses to an increasing statutory retirement age.

### 2.1. Employees' Responses to Increasing Statutory Retirement Ages

First, emotional responses indicate one's personal involvement with a changing context [23]. Because an increasing statutory retirement age directly impacts employees' prospects for retirement timing and has potential financial ramifications, it is likely to invoke emotional responses. The prospect of prolonged employment can be assessed along different dimensions leading to different types of emotions, such as happiness and joy, but also anger and fear or concern on the part of the individual worker, who may worry whether they will be able to keep on working—physically and mentally—till their retirement age. Individuals will assess events according to their individual goals, to their beliefs and values, and in the light of their own potential for coping with the situation [23].

Second, cognitive responses to the changes in the statutory retirement age can be defined as the mental assessments people make when they reflect on the implications of this change and the possible ways of dealing with it. These thoughts might be related to assessing the opportunities for alternative jobs, thinking of changing the direction in a career within or outside the current organization. Cognitive responses might indicate an awareness that adjustments in behavior might be needed to be able to reach the increased retirement age in satisfactory mental and physical health and increase the person-job fit.

Third, behavioral responses to the prospect of a higher retirement age than foreseen refer to actions that workers carry out to increase their work ability (for instance, by investing in skills) and to prepare for the possible future challenges of an extended working life, for instance, by voluntary seeking a demotion or by negotiating a work load or a set of tasks that seem manageable [24]. These behaviors indicate that workers increase their agency of their future career instead of waiting for something to happen.

Emotions, cognitions, and behaviors interact in complex ways. Emotions may encourage or discourage behavior. Cognitive responses about a lack of opportunities to extend the working life in a satisfactory manner might reduce proactive behaviors and may at the same time increase negative emotions. In this article, we assume that individuals may vary in their emotional reactions, the cognitive responses, and their proactive behaviors in reaction to the prospect of a higher statutory retirement age and that the restrictive circumstances people experience in different dimensions play an important role in understanding heterogeneity in employees' responses.

### 2.2. Predictors of Employees' Responses

Employees' use of different strategies in response to changes in the external context is likely to be affected by the resources and restrictions in different dimensions relevant to longer working lives. Retirement age reforms that stimulate longer working lives will signal to employers how to behave and stimulate them to develop practices to support workers' ability for prolonged employment [25]. This perspective of macrolevel impacts on organizational practices has been explored and conceptualized in organizational and institutional theories [26, 27]. Furthermore, retirement age reforms are likely to influence social norms about the appropriate timing of retirement. We formulate a broad *age-inclusivity hypothesis* predicting that workers who perceive their organizational and social contexts to align with the macrolevel drivers for longer working lives have less negative emotions and more proactive cognitive and behavioral involvement in prolonged sustainable careers [25]. Here, we identify three elements of the age-inclusivity context that are deemed particularly important. These concern potentially supportive circumstances in the labor market (low levels of age discrimination), within the employing organization (access to accommodative HR facilities), and in individuals' social networks (supportive social norms for longer working lives). In addition, individuals' responses may also be related to individual-level resources.

First, older workers' experiences of age discrimination in the labor market can influence how people respond to having to work longer. Studies show how (the perception of) age discrimination can lead to decreased employee engagement [18] and decreased performance [28]. Links to a lower desired retirement age have also been found [29, 30], although these results are mixed [18]. With regard to employees' responses, experiencing age discrimination is likely to invoke feelings of inadequacy and thus to spark negative emotional feelings of having to deal with unfair treatment for a longer time. Experiencing age discrimination may not lead to more proactive cognitive and behavioral responses because individuals may feel these are meaningless, as they cannot control their age [31].

Second, organizations may influence their employees' responses through their approach in facilitating and stimulating lifelong learning on the job and reaching their retirement in good health. In particular, HR facilities that may accommodate prolonged employment, such as additional leave, the possibility to reduce work load, or take part-time retirement, can increase employees' engagement and commitment [19] and help older workers sustain their performance and make prolonged employment more attainable [32]. Indeed, Shacklock and Brunetto [33] and Moen and colleagues [34] show that HR facilities that give employees more flexibility in their working arrangements increase employees' intentions and expectations to work longer. With regard to employees' responses, we expect that employees who work in organizations with more easily accessible accommodative HR facilities have fewer negative emotional reactions but more proactive cognitive and behavioral responses that increase their sustainable employability.

Third, social norms about working at later ages may affect how individual employees respond to having to work longer. Social norms can be seen as unwritten rules about the (un)acceptability of behavior in certain social contexts, which may influence individuals either through internalization of the social norms or the threat of social sanctions if norms are not respected [35]. In the context of longer working lives, social norms may exist about the timing of retirement, the acceptability of retiring before or after statutory retirement age, and making a career switch in light of remaining employable [36–38]. Social norms exert their influence through the threat of informal social punishment, such as dismissive attitudes, gossiping, or social exclusion of the norm transgressor [35]. People generally want to avoid such punishments and are therefore expected to comply with the norms in their social networks. We therefore expect that employees that perceive social norms about prolonged employment in their direct social networks to be more restrictive, that is, more accepting about early retirement, but less accepting of working beyond statutory retirement age or making a career switch, are more likely to have negative emotional reactions but less likely to respond with proactive behavior to make prolonged employability attainable.

In addition to contextual factors that might support longer working lives, employees with different demographic and socioeconomic characteristics may differ in their emotional, cognitive, and behavioral responses to an increasing retirement age. These individual-level factors may indicate resources showing a better labor market position and therefore a higher capacity to prepare for prolonged employment. Moreover, these workers may also be more knowledgeable of the extent of increase in the statutory retirement age. We refer to this as the *individual resources hypothesis*: employees with more resources (i.e., higher level of education, higher income, better health, and having a spouse) are expected to have less negative emotions and more proactive cognitive or behavioral responses to the increasing statutory retirement age. Two demographic characteristics, sex and age, are included as control variables. Regarding one's age, it is difficult to predict the relation with responses to an increasing statutory retirement age. One the one hand, those further away from the statutory retirement age are confronted with larger increases in the retirement age, warranting stronger responses, while on the other hand, retirement is further away, which may mitigate negative emotions and the need for cognitive and behavioral responses.

## 3. Methods

### 3.1. Data

Data were collected in February 2020 with a survey among participants of the LISS (Longitudinal Internet Studies for the Social Sciences) panel, which is a representative sample of the Dutch population (for more information, see www.lissdata.nl). The panel consists of approximately 7,500 individuals in 5,000 households. The panel is based on a true probability sample of households drawn from the population register by Statistics Netherlands. Households that could not otherwise participate are provided with a computer and internet connection. Panel members complete online questionnaires every month of about 15 to 30 minutes in total and are paid for each completed questionnaire. For this study, a subsample of 1,997 people aged 40 to 66, whose main activity was paid work, was drawn from the panel. 1,572 participants responded, for a response rate of 79%. Because we are only interested in how people working in organizations respond, we exclude self-employed and family workers and focus solely on employees (87% of respondents). Four cases with missing values on all dependent variables were removed. This leaves 1,351 cases for analysis. There was no item nonresponse in the data, except for the variables on individual net income (5.5% missing values) and health (14.1% missing values). Multiple regression imputation was used to impute missing values on those variables [39]. This did not change substantively the results of the study but was done to obtain the most accurate results. The data can be considered representative for the population of Dutch individuals aged 40 to 66 working in organizations.

### 3.2. Measures

#### 3.2.1. Dependent Variables

The dependent variables in this study are latent constructs of employees' emotional, cognitive, and behavioral responses to an increasing statutory retirement age. The three latent dependent variables are each constructed from three survey items and treated as interval variables, which is a standard approach in structural equation modeling.

In the survey, employees were first presented their expected statutory retirement age, which is based on their date of birth and current projections about the life expectancy at age 65. Then, they were asked “When you think about working until your statutory retirement age,…,” after which multiple items were presented, to which respondents were asked to answer on a scale from “Not at all” (1) to “To a large extent” (4). These items underlie the latent constructs that form the dependent variables in the structural equation model.

Emotional responses were measured with the items “Are you worried about being physically able to do so?”, “Are you worried about being mentally able to do so?”, and “Are you angry?” Cognitive responses were measured with the items “Do you think about taking a different job for the last phase of your career?”, “Do you think about how you can keep meeting the requirements for your current job?”, and “Do you think about how to keep your work pleasurable?” Behavioral responses were measured with the items “Do you keep up to date with new developments in your current work?”, “Do you maintain a healthy work-life balance?”, and “Do you pay more attention to healthy living and working?”

#### 3.2.2. Independent Variables

First, *experiences of age discrimination* were assessed with the question “Have you, in the last five years, ever felt discriminated on the labor market because of your age?”, with answering options “Yes” (1) and “No” (0).

Second, the *accessibility of accommodative HR facilities* was measured with the following question: “How easy is it in your organization to use the following HR facilities?”, after which eight HR facilities were presented that are typically identified as being accommodative in the literature (e.g., [32, 40]), namely, long-term leave, ergonomic support, career interview, flexible working hours, working from home, alleviation of job tasks, working part-time, and demotion. Respondents answered on a scale from “Not possible” (1) to “Very easy” (4). Factor analysis revealed there was one underlying factor, and Cronbach's *α* was high (0.81). These eight items were implemented in the structural equation model as a latent construct representing the accessibility of accommodative HR facilities, to account for measurement error.

Third, to assess the extent to which employees expected to be subjected to *social norms* of their surroundings, we asked the question “How do you think that people in your surroundings would react if…,” with the following more specific items: “You would continue to work after your statutory retirement age?”, “You would make a career switch in the next year?”, and “You would take early retirement and stop working three years before your statutory retirement age?” Respondents answered how they thought people in their surroundings would react on a scale from “Very negatively” (1) to “Very positively” (5).

Finally, a number of individual-level factors were included. Their *age* was determined based on their date of birth and ranged from 40 to 66 due to the study's design focusing on mid- to late-career workers. Respondent's *sex* was also taken from the background information provided by the panel. Their *educational level* was determined by their highest diploma and recoded into International Standard Classification of Education (ISCED) classes, where “Low” stands for primary or lower secondary education, “Medium” stands for upper secondary or postsecondary nontertiary education, and “High” stands for tertiary education. Respondents provided their individual average monthly net income in euros, which was transformed to their *average monthly net income in thousands of euros* for ease of interpretation. They provided their self-rated *health* by answering the question “How would you describe your health, generally speaking?”, with answering options from “Poor” (1) to “Excellent” (5). Finally, *marital status* was taken from the background information of the panel, including the categories “Never married,” “Married,” “Divorced,” and “Widowed.”

### 3.3. Analytic Strategy

**Figure 1:**
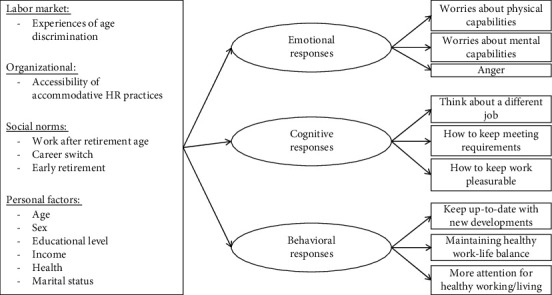
Visual representation of the full structural equation model.

First, we test our hypothesized categorization of employees' responses to the increasing statutory retirement age with a confirmatory factor analysis to assess construct validity. We compare a 3-factor structural equation model, with the proposed categories of emotional, cognitive, and behavioral responses modeled as latent factors, to a 1-factor model. We assess model fit by comparing the common model fit statistics root mean square error of approximation (RMSEA), Aikake information criterion (AIC), Bayesian information criterion (BIC), comparative fit index (CFI), and the Tucker-Lewis index (TLI). Next, to test the relationships between the independent variables and the dependent latent constructs, we estimate a full structural equation model with the three latent factors determined by their respective items, and all independent variables regressed on these latent constructs. This model is visually presented in Figure 1. Below, we present the standardized results, or beta coefficients, of the structural equation model. The beta coefficients can be interpreted as the number of standard deviations change in the dependent variable per standard deviation increase in the independent variable.

## 4. Results

### 4.1. Descriptive Statistics

Table 1 presents the full answering distributions for the measures underlying the latent constructs of emotional, cognitive, and behavioral responses of employees to an increasing statutory retirement age. It is noteworthy that concerns about physical and mental fitness until the increased retirement age are approximately equally distributed, with approximately half of the respondents being worried to a moderate or large extent, and 28% of respondents very angry about the increased retirement age. This is substantially lower than 45% of respondents being “very or extremely angry about the later retirement age” by Van Solinge and Henkens [10] (p.277). This difference is most likely due to differences in populations; while we focus on workers aged 40-66, they focused on workers aged 60-65, who were thus immediately affected by the policy changes. Also, while about two-thirds of respondents think about how they can keep meeting the requirements of their job or how to keep their work pleasurable to some extent, only about four in ten respondents have considered taking another job in the last phase of their career.  Table 2 presents descriptive statistics for all dependent and independent variables.

### 4.2. Multivariate Analysis

To assess the construct validity of the latent response measures, we compare the three-factor model to a one-factor model where all response measures load on a single latent response factor. Goodness-of-fit statistics indicate that the three-factor model (RMSEA = 0.06; AIC = 30,610; BIC = 30,766; CFI = 0.97; TLI = 0.95) is a significantly better fit to the data than the one-factor model (RMSEA = 0.19; AIC = 31,802; BIC = 31,942; CFI = 0.63; TLI = 0.51). Standardized factor loadings for the items underlying the latent constructs in the three-factor model are presented in Table 2.

Table 3 presents the results of the structural equation model estimating the effects on employees' emotional, cognitive, and behavioral responses to an increasing statutory retirement age. Employees that have experienced age discrimination in recent years are more likely to have negative emotional reactions to the increasing statutory retirement age. They are also more likely to think about making changes to their job or way of working, but not more likely to also translate this into actions that facilitate a longer working life. Next, employees of organizations where accommodative HR facilities are more easily accessible are less likely to have negative emotional and cognitive responses but more likely to adjust their behavior to facilitate later retirement than employees of organizations where such accommodative HR facilities are not offered or not easily accessible.

Social norms are also related to employees' responses to an increasing statutory retirement age. Employees whose social environment is more supportive of continuing to work after statutory retirement age are less likely to have negative emotional or cognitive responses, although there is no relation to behavioral responses. A more supportive social environment regarding making a career switch within a year is related to increased thinking about sustaining one's employability and to making behavioral responses that facilitate prolonged employment. A social environment that is more supportive of taking early retirement several years before the statutory retirement age is related to increased negative emotional responses but also to thinking about sustaining one's employability and behavioral responses to facilitate longer working. The effect on negative emotional responses is strongest though, perhaps because the social environment reinforces a belief that early retirement should be possible, and increasing the retirement age is unfair [10].

These findings indicate some support for our age-inclusivity hypothesis, which stated that workers who perceive a mismatch between their organizational and social contexts and the overarching policy goal of longer working lives would experience more negative emotions and display less proactive cognitive and behavioral responses to facilitate their prolonged employment. For example, while experiencing age discrimination is related to more negative emotions, it is also related to increased thinking about how to remain employable, but not to behavior. The hypothesis is mostly supported regarding accessibility of accommodative HR facilities in the employing organization, which is related to fewer negative emotions and increased behavior to facilitate longer working lives, but also to slightly less cognitive engagement with prolonged employment.

With regard to personal factors, a higher age is related to fewer negative emotional and cognitive responses and only slightly higher behavioral responses to the increasing statutory retirement age. A robustness check showed that the age effect on emotional and cognitive responses was most strongly negative for people aged over 60, whereas those aged 40 to 59 differ little in their emotional and cognitive responses, suggesting late-career emotional and cognitive, but not behavioral, disengagement [41]. There were no signs of potential curvilinear relationships between age and employees' responses.

There is also a strong socioeconomic gradient to the responses offering partial support for the individual resources hypothesis: employees with a higher education are least likely to have negative emotional responses but most likely to have cognitive and behavioral responses to an increasing retirement age. Employees with medium and lower educational levels are equally likely to have negative emotional and cognitive responses, but those with a low educational level are less likely than those with a medium educational level to respond with behavior to facilitate prolonged employment. Additional analysis with interaction effects of education and other independent variables (not reported) revealed that especially higher educated employees have fewer negative emotional and more proactive behavioral responses when accommodative HR facilities are easily accessible, while those HR facilities have lower impact on lower educated employees.

Employees with a higher net income are also less likely to have negative emotional responses than those that have a lower income. There are no effects of net income on cognitive and behavioral responses. Finally, a better health status is related to fewer negative emotions and thoughts about prolonged employment but also increased behavior to facilitate longer working lives.

## 5. Discussion and Implications

In this study, we examined the different ways in which mid- and late-career employees respond to increasing statutory retirement ages. Rather than focusing on responses in a single dimension, we conceptually distinguished three dimensions of responses: negative emotional reactions, cognitive engagement with prolonged employment, and proactive behaviors that facilitate longer working lives. We studied how potential restrictions in the labor market, within the employing organizations, and in employees' social environments are associated with the level of emotional, cognitive, and behavioral responses to a higher statutory retirement age. We hypothesized that (1) age inclusivity in organizations and society at large is associated with less negative emotional but more proactive cognitive and behavioral responses to higher statutory retirement ages and (2) employees with more individual-level resources, such as a higher education and better health, have a better labor market position to prepare for prolonged employment and therefore less negative emotional and more proactive cognitive and behavioral responses to an increasing statutory retirement age.

The results show that when employees have access to accommodative HR facilities in their organizations and the perceived social norms regarding prolonged employment are aligned with the overarching societal goal of extending working lives, employees show more signs of adaptive responses. Furthermore, employees who have no experiences with age discrimination are adjusting easier to the increasing statutory retirement age. However, when the organizational and social contexts are misaligned with the overarching policy goal, employees display higher levels of negative emotions and a lower level of behavioral response. The relationship with cognitive response is more mixed. This research shows the importance of supportive contexts at different levels—societally, in organizations, and in individuals' own lives—for policy changes such as increasing the statutory retirement age to be effective [8].

In addition, we found important socioeconomic differences in employees' responses. Highly educated employees were least likely to display negative emotional responses but most likely to show cognitive engagement or proactive behaviors that could lead to successful continued employment and a higher retirement age. Employees with a low or medium educational level, on the other hand, were more likely to display negative emotions and did not think about ways to sustain their employability as much as higher educated employees. Lower educated employees in particular were least likely to behaviorally adjust to the prospect of prolonged employment. Also, employees in poor health have much more difficulty adjusting to higher retirement ages. Poor health evokes strong negative emotions and worries in response to increases in the statutory retirement age, without increasing proactive behavioral engagement supportive of a higher retirement age. This may have long-term implications, as a failure to adapt to the prospect of a longer working life could lead to unemployment and a more precarious financial situation later in life.

Several limitations of this study should be acknowledged. First, while a strength of our approach is to simultaneously consider employees' responses in different dimensions, emotional, cognitive, and behavioral, the measures underlying the latent constructs were limited in their scope. For example, potential positive emotions, such as gratitude or excitement, were not included in our measure. Our construct of behavioral responses was limited to actions that may facilitate longer working in the current job, but did not include employees' behaviors that were focused on another job in the last phase before retirement, although the cognitive measure showed this to be an unlikely occurrence. In addition, the scope of independent variables was limited, and we could not test how, for example, HR facilities for training or early retirement were related to employees' responses. Second, we used cross-sectional data to assess the relationships between the variables in this study, while in reality, responding to changing contexts is a continuous and dynamic process. This implies that although we specified the changing context in our data collection and conceptualization for this study, we cannot ascertain that this is the causal driving force for employees' responses. Furthermore, due to the cross-sectional data, we are unable to test how emotional, cognitive, and behavioral responses are dynamically interrelated and potentially affect each other. Third, we analyzed data from a single country, the Netherlands, and results may not necessarily translate or be generalizable to other countries, especially those with more moderate increases of the statutory retirement age.

To adjust to the long-term challenges of an aging workforce, several countries have reformed their pension system by linking the statutory retirement age to the development of life expectancy. Next to the Netherlands, a link between life expectancy and statutory retirement age has recently been made in countries such as Denmark, Finland, Italy, and Portugal [1]. In these countries, statutory retirement ages are projected to reach much higher levels than ever before, since life expectancy is expected to increase further in the decades to come [42]. This study is one of the first to look at how employees respond to these reforms. The results suggest that adverse emotional, cognitive, and behavioral responses from workers are much more likely in organizational and societal contexts that do not support these longer working lives. This result underscores that reforms of the public pension system as induced by many governments need to be tied to broader reforms of the labor market towards inclusion of older workers to be truly effective.

## Figures and Tables

**Table 1:** Employees' responses to increasing statutory retirement ages (*N* = 1,351).

	Not at all	To a small extent	To a moderate extent	To a large extent
*Emotional*				
Worried about physical capabilities	25%	31%	24%	20%
Worried about mental capabilities	26%	30%	27%	17%
Angry	29%	24%	19%	28%
*Cognitive*				
Thinking about a different job in the last phase of career	60%	21%	13%	6%
How to keep meeting requirements of current job	35%	33%	24%	8%
How to keep work pleasurable	32%	32%	27%	9%
*Behavioral*				
Keep up-to-date with new developments in job	13%	23%	43%	22%
Maintaining a healthy work-life balance	9%	22%	43%	26%
Pay more attention to healthy living and working	11%	28%	45%	16%

**Table 2:** Descriptive statistics (*N* = 1,351).

	Mean or %	SD	Factor loadings
Dependent variables			
*Emotional*			
Are you worried about being physically able to do so?	2.38	1.06	0.88
Are you worried about being mentally able to do so?	2.35	1.05	0.87
Are you angry?	2.45	1.18	0.60
*Cognitive*			
Do you think about taking a different job for the last phase of your career?	1.65	0.93	0.41
Do you think about how you can keep meeting the requirements for your current job?	2.05	0.95	0.78
Do you think about how to keep your work pleasurable?	2.13	0.97	0.78
*Behavioral*			
Do you keep up to date with new developments in your current work?	2.75	0.94	0.43
Do you safeguard your work-life balance?	2.87	0.91	0.64
Do you pay more attention to healthy living and working?	2.66	0.88	0.76
Independent variables			
*Labor market*			
Experienced age discrimination	15.39%		
*Organizational*			
Accessibility of accommodative HR practices	2.45	0.59	
*Social norms*			
Continuing to work after statutory retirement age	3.00	0.87	
Making a career switch within a year	3.27	0.87	
Taking early retirement	3.62	0.78	
*Personal factors*			
Age	52.68	7.21	
Sex			
Male	50.85%		
Female	49.14%		
Education			
Low	18.36%		
Medium	41.15%		
High	40.49%		
Net monthly income (^∗^€1000)	2.18	0.98	
Health	3.15	0.69	
Marital status			
Never married	21.76%		
Married	62.69%		
Divorced	13.92%		
Widowed	1.63%		

**Table 3:** Standardized structural equation model results of the effects of labor market, organizational factors, social norms, and individual-level factors on emotional, cognitive, and behavioral responses to increasing statutory retirement ages (*N* = 1,351).

		Emotional			Cognitive			Behavioral		
		Beta		SE	Beta		SE	Beta		SE
*Labor market*										
Experienced age discrimination		0.08	∗∗	0.03	0.13	∗∗∗	0.03	0.05		0.03
*Organizational*										
Accessibility of accommodative HR facilities		-0.19	∗∗∗	0.03	-0.09	∗	0.04	0.20	∗∗∗	0.04
*Social norms*										
Continuing to work after statutory retirement age		-0.21	∗∗∗	0.03	-0.09	∗	0.03	-0.04		0.04
Making a career switch within a year		0.03		0.03	0.10	∗∗	0.03	0.10	∗	0.04
Taking early retirement		0.16	∗∗∗	0.03	0.08	∗	0.03	0.09	∗∗	0.03
*Individual-level factors*										
Age		-0.12	∗∗∗	0.03	-0.13	∗∗∗	0.03	0.08	∗	0.03
Sex (ref. = male)	Female	0.00		0.03	0.02		0.03	0.05		0.04
Education (ref. = medium)	Low	0.00		0.03	-0.04		0.03	-0.09	∗	0.04
	High	-0.06	∗	0.03	0.10	∗∗	0.04	0.11	∗∗	0.04
Net monthly income (∗€1000)		-0.09	∗∗	0.03	0.05		0.04	-0.07		0.04
Health		-0.18	∗∗∗	0.03	-0.13	∗∗∗	0.03	0.11	∗∗	0.03
Marital status (ref. = never married)	Married	0.01		0.03	-0.03		0.04	-0.01		0.04
	Divorced	-0.03		0.03	-0.01		0.04	-0.02		0.04
	Widowed	0.01		0.03	0.00		0.03	0.00		0.03

^∗^ *p* < 0.05; ^∗∗^*p* < 0.01; ^∗∗∗^*p* < 0.001. Note: covariances between the latent dependent variables are as follows: emotional∗cognitive (0.51; SE = 0.03; *p* < 0.001); emotional∗behavioral (0.10; SE = 0.04; *p* < 0.01); cognitive∗behavioral (0.37; SE = 0.04; *p* < 0.001).

## Data Availability

Data were collected through the LISS (Longitudinal Internet Studies for the Social Sciences) panel. The panel can be accessed at https://www.dataarchive.lissdata.nl/.
